# Novel bioinformatic classification system for genetic signatures identification in diffuse large B-cell lymphoma

**DOI:** 10.1186/s12885-020-07198-1

**Published:** 2020-07-31

**Authors:** Wei Zhang, Li Yang, Yu’ Qi Guan, Ke’ Feng Shen, Mei’ Lan Zhang, Hao’ Dong Cai, Jia’ Chen Wang, Ying Wang, Liang Huang, Yang Cao, Na Wang, Xiao’ Hong Tan, Ken He Young, Min Xiao, Jian’ Feng Zhou

**Affiliations:** 1 Department of Hematology, Tongji Hospital, Tongji Medical College, Huazhong University of Science and Technology, Wuhan, 430030 Hubei Province P.R. China; 2 Department of Hematology/Oncology, Guangxi Medical University Cancer Hospital, No. 71 Hedi Road, Nanning, Guangxi 530021 P.R. China; 3 Department of Pathology, The University of Duke, Durham, North Carolina USA

**Keywords:** DLBCL, Sequencing, Random forest, Classification, Signature

## Abstract

**Background:**

Diffuse large B-cell lymphoma (DLBCL) is a spectrum of disease comprising more than 30% of non-Hodgkin lymphomas. Although studies have identified several molecular subgroups, the heterogeneous genetic background of DLBCL remains ambiguous. In this study we aimed to develop a novel approach and to provide a distinctive classification system to unravel its molecular features.

**Method:**

A cohort of 342 patient samples diagnosed with DLBCL in our hospital were retrospectively enrolled in this study. A total of 46 genes were included in next-generation sequencing panel. Non-mutually exclusive genetic signatures for the factorization of complex genomic patterns were generated by random forest algorithm.

**Results:**

A total of four non-mutually exclusive signatures were generated, including those with *MYC*-translocation (*MYC*-trans) (*n* = 62), with *BCL2*-translocation (*BCL2*-trans) (*n* = 69), with *BCL6*-translocation (*BCL6*-trans) (*n* = 108), and those with *MYD88* and/or *CD79B* mutations (MC) signatures (*n* = 115). Comparison analysis between our model and traditional mutually exclusive Schmitz’s model demonstrated consistent classification pattern. And prognostic heterogeneity existed within EZB subgroup of de novo DLBCL patients. As for prognostic impact, *MYC*-trans signature was an independent unfavorable prognostic factor. Furthermore, tumors carrying three different signature markers exhibited significantly inferior prognoses compared with their counterparts with no genetic signature.

**Conclusion:**

Compared with traditional mutually exclusive molecular sub-classification, non-mutually exclusive genetic fingerprint model generated from our study provided novel insight into not only the complex genetic features, but also the prognostic heterogeneity of DLBCL patients.

## Background

Diffuse large B-cell lymphoma (DLBCL) is the most prevalent lymphoid malignancy in adult patients, comprising 30–40% of non-Hodgkin lymphomas [1]. Although durable remissions can be achieved in a substantial proportion of patients after chemoimmunotherapy with rituximab, cyclophosphamide, doxorubicin, vincristine, and prednisone (R-CHOP), over 30% of cases develop refractory or relapsed disease [2]. Previous studies have revealed that DLBCL is a genetically heterogeneous disorder with considerable gene mutations, copy number (CN) alterations, and structural variants [3–5]. Understanding the molecular basis of this heterogeneity may facilitate individualized management strategies.

Researchers have focused on developing a robust algorithm to discover distinct subsets and subclassify DLBCLs. In 2000, based on gene expression profiling results, Alizadeh et al. identified two molecularly distinct forms of DLBCL, germinal center B-cell-like (GCB) and activated B-cell-like (ABC), representing different stages of B-cell differentiation [6]. Nevertheless, according to the cell-of-origin (COO) classification, approximately 10% ~ 20% of DLBCLs remain unclassified, and the molecular pathogenesis of DLBCL remains obscure. The rapid development of next-generation sequencing (NGS) technology has revealed accumulated recurrent genetic alterations, which have improved our understanding of the genetic landscape of DLBCL. However, these genomic studies had largely focused on single types of alterations. In 2018, Schmitz et al. studied tumor specimens from 574 patients with DLBCL [4]. By developing a subclassified algorithm based on coding region mutations, CN variations, and structure variations (SVs), they further identified four distinct genetic subtypes of DLBCL, which included BN2 (based on *BCL6* fusions and *NOTCH2* mutations), N1 (based on *NOTCH1* mutations), MCD (based on the co-occurrence of *MYD88*^L265P^ and *CD79B* mutations), and EZB (based on *EZH2* mutations and *BCL2* translocations). Chapuy et al. also carried out a comprehensive genetic analysis of 304 primary DLBCLs and identified five subgroups of DLBCLs with prominent genetic features (C1–C5), [5]. These studies provided us with a novel roadmap for an actionable DLBCL classification for precision-medicine-based strategies in DLBCL.

Although several specific subgroups, such as primary DLBCL of the central nervous system (PCNSL), primary mediastinal (thymic) large B-cell lymphoma (PMBL), primary cutaneous DLBCL, leg type (PCDLBCL-LT), high-grade B-cell lymphoma, not otherwise specified (HGBL, NOS), and HGBL with *MYC* and *BCL2* and/or *BCL6* translocations (HGBL-DH/TH), are all well-defined entities, they share clinicopathologic features and genetic alterations with DLBCL, NOS according to previous studies [7–10]. In fact, they belong to a disease spectrum rather than discrete entities. In 2018, Scott et al. defined a clinically and biologically distinct subgroup of tumors within GCB DLBCL characterized by a gene expression signature of HGBL-DH/TH-*BCL2* [11]. Simultaneously, Westhead et al. also defined a molecular high-grade (MHG) group by applying a gene expression–based classifier [12]. Chapuy et al. also mentioned in their study that molecular heterogeneity existed within the C3 subgroup [5]. Their studies suggest that beyond current definitions of double- and triple-hit DLBCLs, more specified molecular subgroups of DLBCL requires further exploration.

In this study, instead of mutually exclusive sub-classification, we determined several non-mutually exclusive genetic signatures for the factorization of complex genomic patterns in a continuous spectrum of B-cell lymphomas based on a random forest algorithm. Using this model, we also presented a single-center primary site-relevant mutational pattern based on the Chinese population. This study aims to develop a novel approach to understand the molecular features, provide a distinctive insight nosologically, and orient targeted therapeutic strategies and prognostic evaluation of the spectrum of large B-cell lymphomas.

## Methods

### Patients and samples

This study was approved by the institutional review board of Tongji Hospital. From June 2008 to September 2018, a cohort of 342 patient samples diagnosed with DLBCL in our hospital were retrospectively enrolled in this study. All cases were reviewed by at least three experienced hematopathologists. Most samples were pretreatment biopsies from de novo cases (*n* = 298), including DLBCL, NOS (*n* = 239), PMBL (*n* = 13), PCNSL (*n* = 7), PCDLBCL-LT (*n* = 6), HGBL, NOS (*n* = 7), and HGBL-DH/TH (*n* = 26). The remainder consisted of relapsed DLBCL, NOS (*n* = 25) after R-CHOP or CHOP-like chemotherapy and transformed follicular lymphoma (tFL) samples (*n* = 19). All samples were obtained from formalin-fixed paraffin embedded (FFPE) tissue. Tumor content was estimated to be at least 30% in all subjects. Genomic DNA was extracted with the GeneRead™ DNA FFPE Kit (Qiagen) according to the manufacturer’s instructions.

DLBCL samples were classified into GCB and non-GCB subtypes by the IHC-based Hans algorithm [13, 14]. Translocations in *MYC*, *BCL2*, and *BCL6* and CN aberrations in *TP53* were examined by performing fluorescent in situ hybridization (FISH). Cases were excluded if COO subtype or FISH findings were not available. Clinical data, including age group of onset, International Prognostic Index (IPI), primary site of lymphomagenesis, chemotherapy regimen, initial response to therapy, overall survival (OS), and progression-free survival (PFS), were collected (Table 1) (Supplementary Table S1). We restrictively included individuals in which biopsy tissue was evaluated as the primary site of lymphomagenesis, according to integrated judgment based on PET-CT scanning, pathological findings, and clinical manifestation [15]. Criteria of response assessment and definition of OS and PFS were followed by the Lugano Classification [16]. Only patients receiving R-CHOP or R-CHOP-like regimens were included in the prognostic analyses. Multivariable Cox proportional hazard regression models were used to evaluate proposed prognostic factors.

**Table 1:** Baseline features of 342 cases included in this study

Parameters	De novo cases (*n* = 298)						Relapsed cases	Transformed cases
	DLBCL, NOS (*n* = 239)	PMBCL (*n* = 13)	PCNSL (*n* = 7)	PCDLBCL-LT (*n* = 6)	HGBL, NOS (*n* = 7)	HGBL-DH/TH (*n* = 26)	DLBCL, NOS (*n* = 25)	tFL (*n* = 19)
Gender								
Male	131	6	6	2	5	9	16	15
Female	108	7	1	4	2	17	9	4
Age of onset								
Median (years)	52	32	59	68	60	50	56	48
COO subtype								
GCB	95	6	0	2	4	23	12	14
Non-GCB	144	7	7	4	3	3	13	5
Type of biopsy tissue								
Nodal	122	0	0	0	5	19	14	15
Extranodal	117	13	7	6	2	7	11	4
IPI at first diagnosis								
0 ~ 1	56	3	1	0	2	5	3	N/A
2 ~ 3	134	8	5	4	2	15	17	N/A
4 ~ 5	49	2	1	2	3	6	5	N/A
First chemotherapy regimen								
R-CHOP or R-CHOP-like	228	13	0	6	7	26	25	9
Other or N/A	11	0	7	0	0	0	0	10

Abbreviations: *DLBCL, NOS* Diffuse Large B cell Lymphoma, Not Otherwise Specified, *PMBCL* Primary Mediastinal B-cell Lymphoma, *PCNSL* Primary Central Nervous System Lymphoma, *PCDLBCL-LT* Primary Cutaneous Diffuse Large B Cell Lymphoma, Leg Type, *HGBL, NOS* High Grade B-Cell Lymphoma, Not Otherwise Specified, *HGBL-DH/TH* High-Grade B-cell Lymphomas harboring rearrangements of MYC and BCL2 and/or BCL6, *tFL* Transformed Follicular Lymphoma, *GCB* Germinal Center B-cell-like, *IPI* International Prognostic Index, *R-CHOP* Rituximab plus Cyclophosphamide, Doxorubicin, Vincristine, and Prednisone;

### Targeted high-throughput sequencing

A total of 46 genes were selected in this study (Supplementary Table S2). Most genes were frequently altered in DLBCL according to data from several previously published large-scale DLBCL cohort studies [3–5]. Additionally, several infrequently mutated genes were also included because they are specifically related to several DLBCL subtypes. In detail, ID3, TCF3, DDX3X are related with double-hit lymphoma [17]/Burkitt lymphoma [18]; XPO1 is related with primary mediastinal large B-cell lymphoma [8]; RRAGC, POU2AF1 are related with Follicular lymphoma [19]; KLF2 is related with marginal zone lymphoma [20]. Besides, in our panel we also selected genes functioning in signaling pathways which are crucial for DLBCL pathogenesis (e.g. FBXW7 for NOTCH pathway; NRAS, KRAS, MAP 2 K1 for MAPK pathway). Using genome build hg19/GRCh37 as a reference, a sequencing panel covering the coding sequences (CDS) within 5 intronic base pairs around exons in 46 genes was designed online (Designstudio Sequencing, Illumina, San Diego, USA). Sequencing libraries were prepared with AmpliSeq™ Library PLUS for Illumina, using 20 ng of input genomic DNA per sample. Library sequencing was performed to 2000× coverage on a NextSeq™ 550 system using an Illumina NextSeq™ 500/550 High Output v2 Kit (Illumina, San Diego, USA). The alignment and variant calling were performed using a DNA Amplicon workflow with default parameters on BaseSpace Sequence Hub (Illumina). Generated variants were further annotated using Annovar [21].

Variant filtering was performed by the following cascade of steps: 1) select exon nonsynonymous or splice donor/acceptor site variants; 2) exclude variants with population frequency > 0.0001 in the gnomAD database unless variant is included as a somatic variant of lymphoid neoplasm in the COSMIC database; 3) exclude variants present in an in-house curated blacklist. The formation of our variant screening blacklist was based on the idea described previously by Schmitz et al. [4] As these false positive variants were presumed to be artifacts generated either by the high throughput sequencing platform itself or due to errors in alignment or annotation of the sequencing reads by the analytical pipeline. Typically, these variants were abnormally prevalent, identified exclusively in specific sequencing platform, and are not recurrent variants included in the major public cancer somatic mutation database (COSMIC database, https://cancer.sanger.ac.uk/cosmic). Therefore, as such variants were unique in our center and there were no universal criteria for identification, the blacklist was built for future rapid and accurate variants’ screening. Detailed variants information included in the blacklist were listed in Supplementary Table S7; 4) exclude variants found in regions with poor coverage; and 5) exclude variants with quality less than 30 or read depth less than 20. For activation-induced cytidine deaminase (AID) somatic hypermutation (SHM) analysis, we additionally selected synonymous variants and variants in intron/UTR regions, and each variant also needed to fulfill the aforementioned criteria from step 2 to step 5 [22].

Sanger sequencing in matched normal DNA was performed if it was available for each missense mutation that passed all preceding filters and met the following conditions: 1) variant allele frequency (VAF) more than 0.40 and less than 0.60; 2) variant was not included in the COSMIC database; 3) variant was included in the gnomAD database. Confirmed germline mutations were excluded in further analyses.

### Fluorescent in-situ hybridization analysis

Interphase fluorescence in situ hybridization (FISH) studies were performed using commercially available probes (Abbott Molecular, Downers, Grove, IL, USA). The LSI IGH/IGHV (14q32), and LSI MYC (8q24) Dual Color, Break Apart Rearrangement Probes were used to detect the rearrangement of BCL2, BCL6 and c-Myc respectively. A 17p13.1 (P53) probe (Vysis, Downers, Grove, IL) was used to detect 17p deletion. Sample preparations and hybridizations were conducted following the manufacturer’s recommendations and 200 cells were analyzed for each probe.

### Bioinformatic algorithm

Artificial Intelligence (AI) is the intelligence manifested by a human-made machine. It usually refers to the ability of a computer to simulate human thought processes in order to mimic human abilities or behaviors. AI not only deals with problems under pre-set rules, but also develops capabilities to generate judgements under new situations through feature identification. The performance of these feature-driven algorithms can improve as they are exposed to more data over time, which is similar to the human learning process. Therefore, such algorithms are named machine learning. Among existing learning methods, random forests are an ensemble learning method for classification or regression that operate by constructing a multitude of decision trees at training time and outputting the classification or regression of the individual trees. In this study, our model was trained using the R package ‘randomForest’. The number of trees was set to 100; all other hyperparameters were set to their default values. Detailed information on the algorithm was described in Supplementary Appendix.

### Statistical analysis

All statistical analyses were evaluated by R v3.5.1. Differences were analyzed using Fisher’s exact test for categorical variables. The significance of the co-occurrence or mutual exclusivity was calculated using a Fisher exact test, for numerous tests, *p* values were FDR - adjusted using Bonferroni method. The Kaplan-Meier method and log-rank test were used for survival analysis. Unless otherwise specified, a two-sided *P*-value < 0.05 was considered statistically significant for all analyses.

## Results

### Mutational profile through next-generation sequencing analysis

**Fig. 1:**
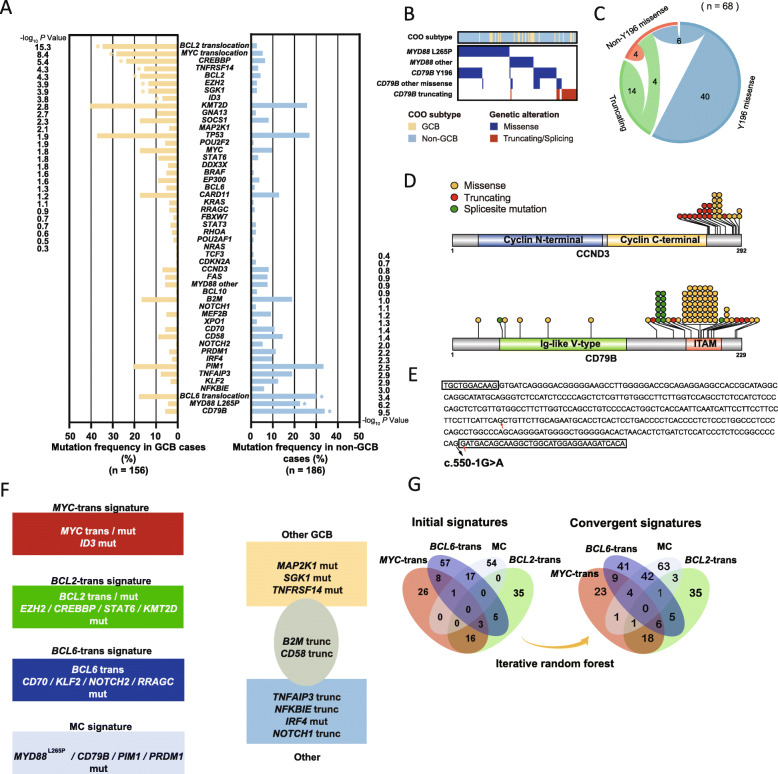
Overview of the genetic features in 342 cases. **a** Frequency of genetic alterations that distinguish the GCB and non-GCB subtypes of 342 cases, sorted by log_10_*P* value for the difference between the two subgroups. **b** The correlation among different types of *MYD88* and *CD79B* mutations. **c** Circos plot depicting the correlation among different types of *CD79B* mutations (Y196 missense, truncating, and non-Y196 missense mutations). **d** Positions and types of somatic mutations encoded in *CCND3* (NP_001751.1) and *CD79B* (NP_000617.1). **e** The sequence of *CD79B* (chr:62007140–62,006,802, *GRCh37*/*hg19*). The black arrow denotes the splice acceptor site mutation c.550-1G > A (NM_000626.4). The red arrow denotes two exposed potential splice acceptor sites. Coding sequences are highlighted by black frames. **f** Genetic alterations that are most related to the *MYC*-trans signature, *BCL2*-trans signature, *BCL6*-trans signature, and MC signature. Recurrent altered genes in GCB and non-GCB cases without our set of genetic signatures were also shown. **g** Venn diagrams describing the difference between cases exhibited initially defined signatures, and cases exhibited extended genetic signatures obtained from a convergence predicted by an iterative random forest algorithm

A total of 1240 candidate variants were identified in 46 genes (Supplementary Table S3). Of 342 cases, 330 (96.5%) harbored at least one mutation. Firstly, as for de novo DLBCL, NOS patients, the most frequently mutated genes were *TP53* (28.9%), *PIM1* (27.2%), *KMT2D* (25.9%), *MYD88* (23.0%), and *CD79B* (22.6%) (Supplementary Fig. S1), and variant frequency of *MYD88* was significantly more frequent compared with relapse DLBCL cases (*P* = 0.014). As for relapse DLBCL cases, *KMT2D* and *KRAS* mutations were significantly more frequent compared with those in de novo cases (*P* = 0.031; *P* = 0.012). Secondly, as for gene functional groups, mutations in genes associated with chromatin remodeling, including *KMT2D* and *CREBBP*, and genes associated with apoptosis resistance, including *BCL2,* were significantly enriched in cases of transformed follicular lymphoma (tFL) in comparison with de novo cases (*P* = 2.20 × 10^− 4^; *P* = 3.51 × 10^− 4^; *P* = 1.93 × 10^− 5^). While immune evasion-related genes, including *B2M*, *CD58*, and *CD70,* were frequently detected in de novo cases (16.3, 12.1, 9.6%) but not in tFL in our cohort (*P* = 0.039; *P* = 0.095; *P* = 0.158). Thirdly, through comparison of mutation gene pattern differences among GCB and non-GCB DLBCL subgroups in all de novo, relapsed, and transformed cases, we determined that the *BCL2* translocation; the *MYC* translocation; and the *CREBBP*, *TNFRSR14*, *BCL2*, *EZH2*, *SGK1*, and *ID3* mutations were significantly more frequent in GCB DLBCL (*P* < 0.001), whereas *CD79B* mutations, the *MYD88*^L265P^ mutation, and the *BCL6* translocation were more common in non-GCB DLBCL (*P* < 0.001) (Fig. 1a).

In addition, in this study we specifically focused on the analysis of *CD79B* mutation pattern. In general, the variant frequency of *CD79B* was relative higher compared with recent related studies [3–5, 23, 24]. In detail, for the typical hotspot variant *CD79B*^Y196^, we found the top co-occurrent mutation with *CD79B*^Y196^ was *MYD88*^L265P^ (*n* = 37) (adjusted *P* value 1.09 × 10^− 9^) in our de novo DLBCL, NOS cases, which was mainly identified in non-GCB subtype (4.5% of GCB cases vs. 22.6% of non-GCB cases, *P* = 5.87 × 10^− 7^) (Fig. 1b). Meanwhile, we found that C*D79B* non-Y196 codon mutations accounted for 41% (33/80) of all *CD79B* mutations. Moreover, through validation Sanger sequencing of tumor and paired normal tissue DNA, we determined several novel hotspot intron splice site mutations, including c.550-1G > A, c.550-1G > C, c.550-3_552del, c.549 + 1G > A, c.549 + 1G > C, and c.540_549 + 1del (Fig. 1d). Focusing on the molecular impact of c.550-1G > A mutation, we subsequently performed RNA sequencing and revealed that this mutation resulted in exposing two novel potential splice acceptor sites, thereby synthesizing two truncating proteins (Fig. 1e). Furthermore, we also found that *CD79B* truncating mutations were mutually exclusive with *CD79B*^Y196^ (adjusted *P* value 0.011). Similar tendency was witnessed for CD79B truncating mutations with *MYD88*^L265P^, while without statistical significance (adjusted *P* value 0.14) (Fig. 1b, Fig. 1c).

### Identification of genetic signatures via iterative random forest (RF) algorithm

In this study, based on targeted sequencing results and FISH findings, we attempted to identify several non-mutually exclusive representative genetic signatures instead of categorizing subjects into several mutually exclusive distinct subgroups. Therefore, we decided to seed our analysis from 5 genetic alterations which participated in the most important cellular signaling pathways in DLBCL pathogenesis, i.e. cellular proliferation (MYC translocation), apoptosis resistance (BCL2 translocation), immune cell differentiation abruption (BCL6 translocation) and activation of inflammation pathway (CD79B ^Y196^ and MYD88 ^L265P^). Moreover, all five genetic alterations were specifically enriched in either GCB or non-GCB subtype DLBCL patients (> 20% positive in GCB or non-GCB DLBCL patients). In addition, these alterations exhibited most distinctive frequencies between GCB and non-GCB DLBCL subtypes by Fisher’s test (Fig. 1a). Thus, using the five main features above, we initially defined four non-mutually exclusive genetic signatures: 1) the *MYC*-trans signature, with *MYC* translocation (*n* = 54); 2) the *BCL2*-trans signature, with *BCL2* translocation (*n* = 59); 3) the *BCL6*-trans signature, with *BCL6* translocation (*n* = 91); and 4) the MC signature, with *MYD88*^L265P^ and/or *CD79B*^Y196^ mutations (*n* = 72) (Fig. 1f).

Among the above-mentioned four signatures, MC signature combined CD79B ^Y196^ and MYD88 ^L265P^ variants as they not only presented as hotspot mutations in DLBCL patients, but also exhibited statistically significant tendency for co-occurrence (adjusted *P* value 1.09 × 10^− 9^). In addition, previous researches also revealed that both variants resulted in constitutive activation of NF-κB signaling pathway [5]. Inspired by the study conducted by R. Schmitz et al., we aimed to evolve and maximize each genetic signature with our set of genetic features while appropriately maintaining the pattern suggested by the initial genetic signature. To alleviate such semisupervised problems, we developed an iterative random forest (RF) algorithm (Supplementary Appendix). The label of each genetic signature among cases gradually propagated and obtained convergence (Supplementary Table S4; Fig. 1g). Additionally, 8 (14.8%), 10 (16.9%), 17 (18.7%), and 43 (59.7%) cases were predicted to exhibit the *MYC*-trans, *BCL2*-trans, *BCL6*-trans, and MC signatures, respectively, suggesting that the initial definition of the MC signature might be conservative. As a result, 252 out of 342 cases (73.7%) were finally confirmed to be associated with at least one genetic signature.

**Fig. 2:**
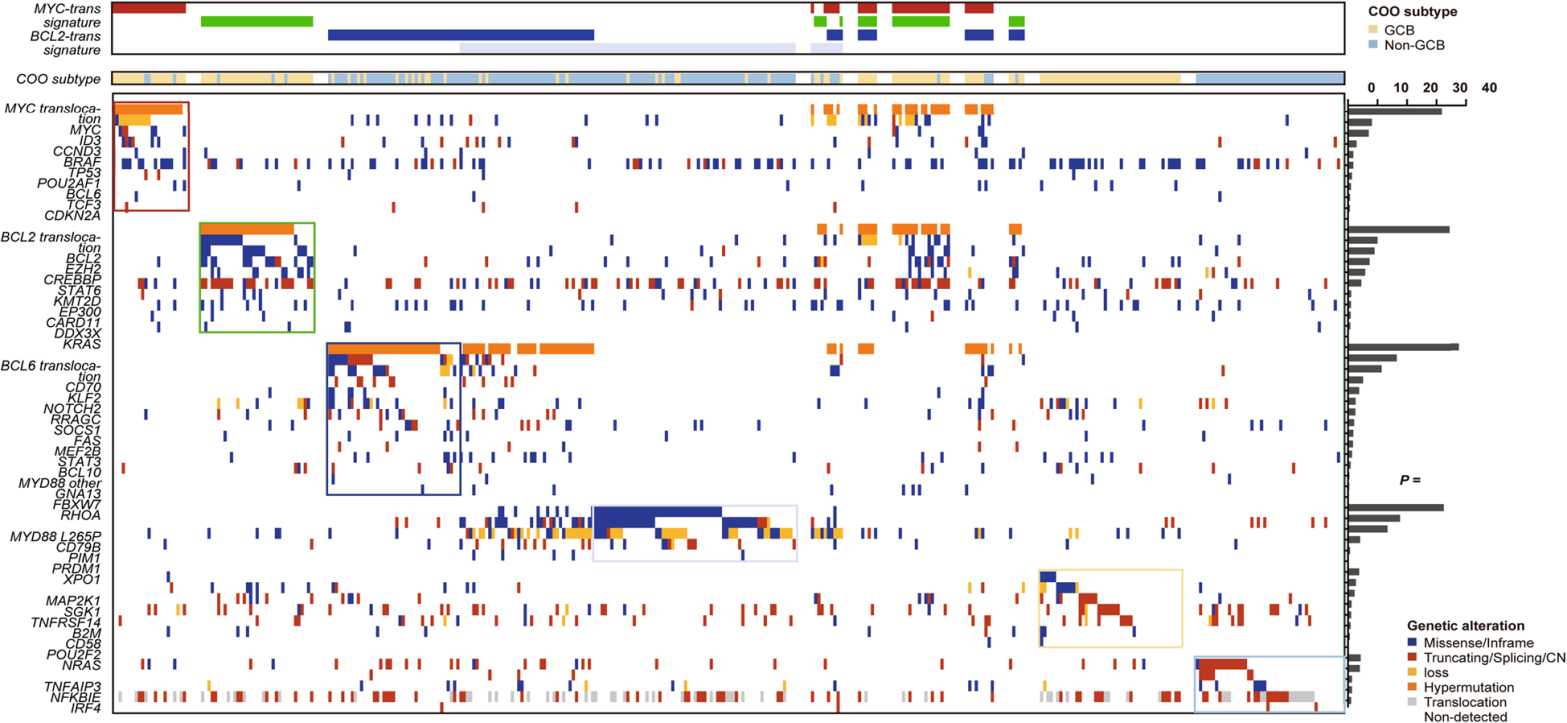
Schematic of the association between genetic alterations and genetic signatures. All 342 cases were clustered and arranged according to the absent/present status of four genetic signatures. We determined the prevalence of each genetic alteration in the following six subsets: 1) cases presented isolated *MYC*-trans signatures, 2) cases presented isolated *BCL2*-trans signatures, 3) cases presented isolated *BCL6*-trans signatures, 4) cases presented isolated MC signatures, 5) GCB cases without any genetic signatures, and 6) non-GCB cases without any genetic signatures. Genetic alterations were thus clustered into six corresponding classes depending on their maximum prevalence among the six subsets. Genetic alterations in the same cluster were ranked by the significance between cases with isolated corresponding genetic signatures and cases without corresponding genetic signatures (or “other GCB”/“non-GCB” vs. the remaining), with log_10_*P* value depicted to the right of the factorized heatmap. Color code of genetic alteration types: missense mutation or in-frame deletion/insertion (blue), truncating mutation, splice donor/acceptor site mutation, or copy number loss in *TP53* (red), SHM (yellow), translocation (orange), and nondetected (gray). COO classification was also indicated above the factorized heatmap

Next, we investigated other genetic mutations statistically associated with one of these genetic signatures. As illustrated in Fig. 2, genetic mutations of each case were combined and clustered within different genetic signatures, and were shown in factorized mutational heatmap. Firstly, *MYC* and *ID3* mutations were associated with the *MYC*-trans signature (*P* < 0.001), and 40% (8/20) of cases with isolated *MYC*-trans signatures harbored mutations in the *ID3*-*TCF3*-*CCND3* pathway. We also recognized that all *MYC* hypermutations were identified in cases with *MYC*-trans signatures (20/20, 100%), while *MYC* non-hypermutations were common in cases with either *MYC*-trans signatures (10/25, 40%) or *BCL6*-trans signatures (15/25, 60%). Secondly, *BCL2*, *EZH2*, *CREBBP*, *STAT6*, and *KMT2D* mutations were significantly related to the *BCL2*-trans signature (*P* < 0.001). Although the *BCL2* mutation was associated with the *BCL2*-trans signature, cases harboring the *BCL2* hypermutation usually implied that they had a combined *MYC*-trans and *BCL2*-trans signature (6/6, 100%). For chromatin modification-associated genes such as *KMT2D* and *CREBBP*, cases harboring co-occurring mutations in *KMT2D* and *CREBBP* generally indicated a *BCL2*-trans signature (21/24, 87.5%). Thirdly, for the *BCL6*-trans signature, the *CD70*, *KLF2*, *NOTCH2*, and *RRAGC* mutations were specifically identified (*P* < 0.001). Although the *CCND3* mutation was more specifically associated with the *MYC*-trans signature (*P* = 0.001), it was also frequent in cases with the *BCL6*-trans signature (16/108, 14.8%). A vast majority of *KLF2* zinc finger mutations (15/22, 68.2%) were identified in cases with *BCL6* translocation (or *BCL6*-tran signature, 21/22, 95.5%), which had not been previously reported. Finally, for the MC signature, in addition to the *CD79B*^Y196^ and *MYD88*^L265P^ mutations, other types of mutations, such as *PIM1* and *PRDM1*, were also significantly related to the MC signature (*P* < 0.001). *XPO1* E571K, a hotspot mutation in chronic lymphocytic leukemia (CLL) and PMBL, was also frequently identified in cases with MC signatures and was usually accompanied by the *BCL6*-trans signature [25, 26].

### Model comparison with classical DLBCL subtype classifier and its prognostic significance

**Fig. 3:**
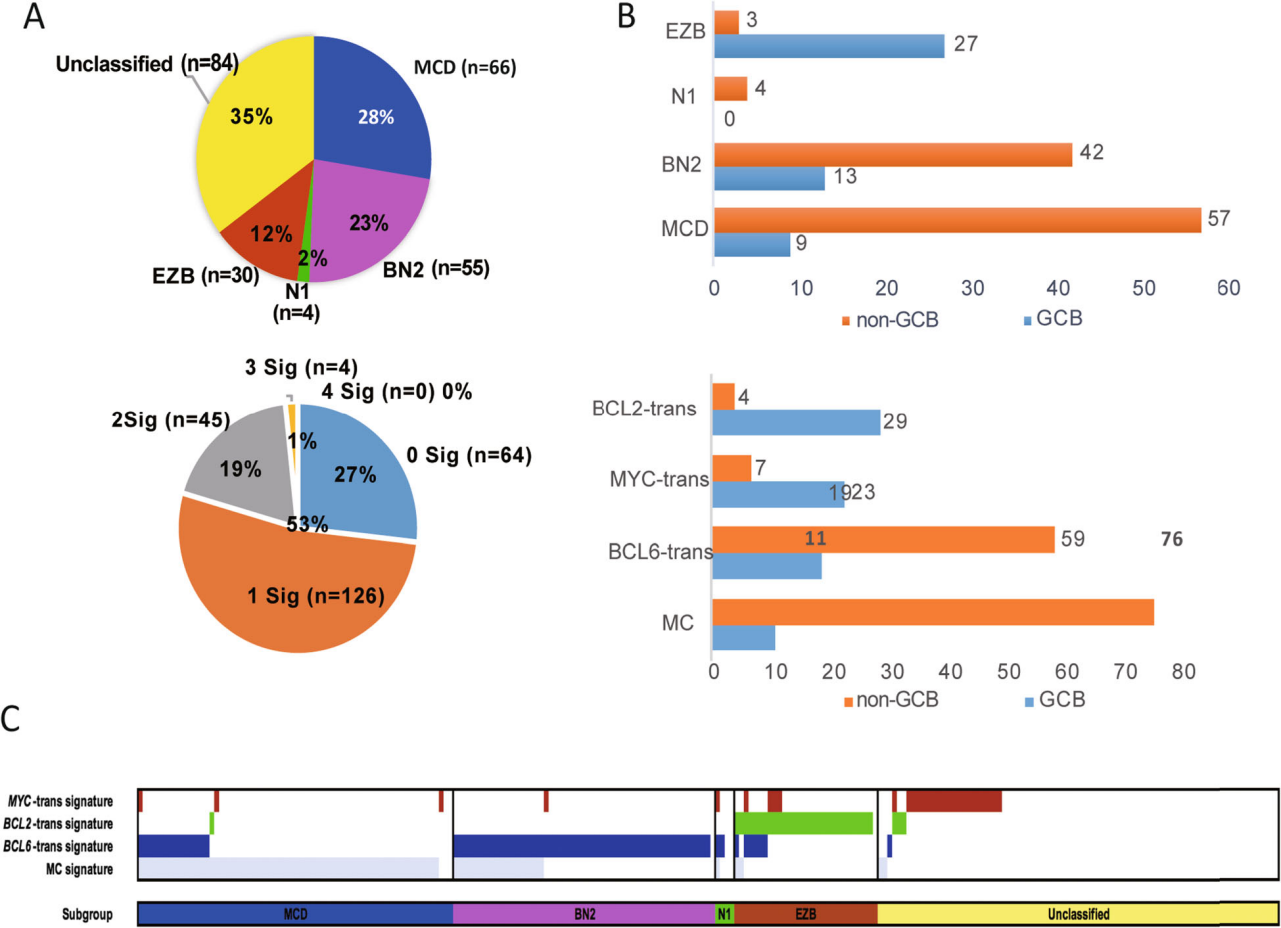
Comparison analysis with mutually exclusive classification model (Schmitz’s model). **a** Patient number and percentage of each subtype classified by traditional mutually exclusive method using Schmitz model (upper) and non-mutually exclusive method using RF algorithm (lower). **b** COO classification of De Novo DLBCL, NOS patients (*n* = 239) grouped by traditional mutually exclusive method using Schmitz model (upper panel) and non-mutually exclusive method using RF algorithm. **c** Classification of De Novo DLBCL, NOS patients (*n* = 239) by traditional mutually exclusive method using Schmitz model and signatures carried by each patient were identified by RF algorithm and shown in upper panel

In order to validate our genetic classification algorithm, we compared our model with the classical DLBCL genetic classifier built from Schimtz et al. [4] for 239 de novo DLBCL NOS cases in our study cohort. As illustrated from Fig. 3a, 65% of all cases (*n* = 155) were successfully classified into four genetic subtypes (MCD *n* = 66, BN2 *n* = 55, EZB *n* = 30, N1 *n* = 4). In comparison, 75% of all cases (*n* = 175) could be classified in at least one signature subtype. COO classification also demonstrated similar type distribution (GCB and non-GCB) between two models (Fig. 3b). As for each genetic subtype of Schimtz et al. (Fig. 3c), the majority of cases within MCD subtype could be grouped in MC-trans signature (63 of 66, 95.4%), and the consistent result was seen in BCL6-trans signature within BN2 subtype (54 of 55, 98.2%) and BCL2-trans signature within EZB subtype (29 of 30, 96.7%). However, in addition to the consistency between two models mentioned above, we did find that a portion of the DLBCL cases within each subtype of Schimtz’s model carried 2 or more signatures. In detail, within MCD and BN2 subtypes, 15 out of 66 (22.7%) and 19 out of 55 (34.5%) patients carried both MC-trans and BCL6-trans signatures, respectively. While in EZB subtypes, 6 out of 30 (20.0%) patients carried both BCL2-trans and BCL6-trans signatures.

To evaluate the prognostic value of our genetic subtype model, we selected all de novo patients with large B-cell lymphoma who received R-CHOP or R-CHOP-like chemotherapy (*n* = 280, maximum follow-up 60 months, median follow-up 26 months). We next constructed a multivariate Cox proportional hazard regression model considering both genetic signatures and IPI scores as variables. The *MYC*-trans signature was the most unfavorable genetic signature, and the *MYC*-trans signature had a hazard ratio (HR) of 2.00 compared with the absented *MYC*-trans signature (OS: *P* = 0.006) (Supplementary Table S5). Those who presented a *BCL2*-trans signature had a relatively favorable 5-year PFS, with a borderline significance (*P* = 0.087). According to the non-mutually exclusive nature of our set of four genetic signatures and several latest research achievements [3, 5, 11, 12, 27], we aimed to explore the differences in prognostic impact for de novo DLBCL cases with various genetic signature numbers. Firstly, in order to exclude the potential influences of confounding factors, especially IPI score, we examined the statistical differences of IPI score group distribution (low 0–1, intermediate 2–3, high 4–5) between groups of patients with varying number of genetic signatures (0-sig, 1-sig, 2-sig, 3-sig). As a result, no statistical differences of IPI level distribution were identified between 0-.

**Fig. 4:**
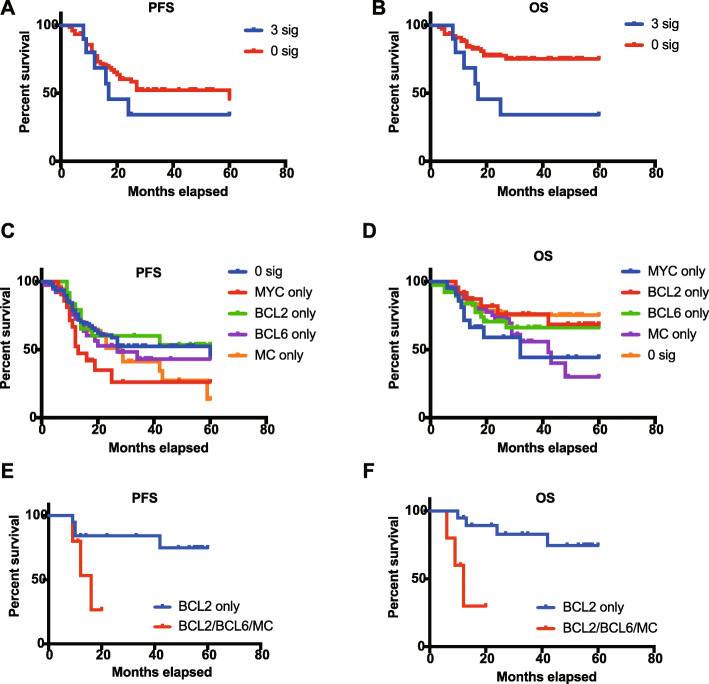
survival analyses of de novo cases that received R-CHOP or CHOP-like chemotherapy. **a-b** Kaplan-Meier plot for 5-year OS and PFS, respectively, according to the signature numbers carried by each case (3 signatures or 0 signature). **c-d** Kaplan-Meier plot for 5-year OS and PFS, respectively. Each case carried a single or no signature. Cases were grouped according to the signature type (MYC-trans, BCL2-trans, BCL6-trans or MC-trans). **e-f** Kaplan-Meier plot for 5-year OS and PFS, respectively. Patients grouped in EZB subtype were classified by signature type (BCL2-trans only or BCL2-trans plus BCL6-trans or MC-trans)

Sig, 1-sig, 2-sig and 3-sig patient groups (*p* > 0.05, Chi-square and Fisher Exact test with Bonferroni adjustment). As reflected by the 5-year OS and PFS time (Fig. 4a-b), we found that individuals carrying three signatures had much worse prognosis than individuals without any genetic signature (OS: *P* = 0.0084; PFS: *P* = 0.3274), while patients with only one genetic signature exhibited no significant difference in prognosis compared with those without any signature (Fig. 4c-d). In addition, further subgroup survival analysis indicated that within EZB subtypes of Schmitz model, patients carrying BCL2-trans plus BCL6-trans or MC-trans signatures exhibited significantly inferior prognosis, compared with patients carrying BCL2-trans signature only (OS: *P* = 0.002; PFS: *P* = 0.039) (Fig. 4e-f). However, no prognostic differences were identified in patients carrying different number of signatures within MCD, BN2 or N1 subgroups. The above findings provided evidence that these non-mutually exclusive genetic signatures exhibited cumulative prognostic influences, and patient heterogeneity still existed in traditional mutually exclusive classification model for DLBCL patients in our cohort, which requires further confirmation in larger multi-center cohort studies.

## Discussion

In this study we retrospectively analyzed NGS sequencing results of DLBCL cohort. Among all panel genes sequenced, we focused on the variant pattern of *CD79B*. In general, variant frequency of *CD79B* was relative higher comparing with reports from other centers, which could be possibly explained with ethnical difference and limited sample number. As has been described in previous reports, the majority of *CD79B* variants including hotspot Y196 clustered in ITAM domain, which were related with NF-κB pathway activation. Our result was consistent with the above findings as the majority of cases bearing *CD79B* mutations were classified into non-GCB subtype. Moreover, it was worth mentioning that in our study we reported for the first time that a series of intron splice-site were identified as recurrent variants in DLBCL patients (e.g. c.550-1G > A, c.550-1G > C, c.550-3_552del, etc.). Our results further indicated that such splice-site variants probably result in CD79B protein truncating, causing CD79B dysfunction in a unique way compared with *CD79B*^Y196^ variant. Furthermore, co-mutation analysis indicated significantly difference compared with classical *CD79B*^Y196^ variant in terms of variant co-occurrence with *MYD88*^L265P^. COO classification also demonstrated differences in DLBCL patients carrying *CD79B* splice-site variants, in comparison of those with *CD79B*^Y196^ variant.

In summary, our results provided evidences that such obviously different mutation pattern of *CD79B* splice-site variants suggested differential impact on DLBCL pathogenesis. However, the exact impact of CD79B truncating protein on the physiological signal transduction of NF-κB pathway and DLBCL pathogenesis calls for further functional study.

Until now, comprehensive studies have revealed that the genetic landscape of DLBCL is heterogeneous, which aids in our understanding of oncogenic mechanisms and provides novel insight into exploring better treatment strategies. To date, Schmitz et al. and Chapuy et al. showed that most DLBCL cases could be subcategorized into several distinct subsets, each of which had unique clinical, molecular and transcriptional characteristics. However, there is still inevitable heterogeneity within each group in their models. Nevertheless, if all expanding factors were taken into consideration, the system would gradually become too complicated to apply in routine clinical scenario. Therefore, in this study, instead of mutually exclusive classification, we aimed to define several non-mutually exclusive genetic signatures to describe and understand the complex molecular features of DLBCL, based on molecular information that was feasible to obtain including *MYC*/*BCL2*/*BCL6* translocations as well as mutation data from a limited panel of genes. In this study, based on previous research methods and results, we preliminarily determined four genetic signatures using a machine learning-based algorithm. In addition, through analysis of prognostic data based on their signature types, we demonstrated unique cumulative prognostic impact based on the number of signatures each patient carry. Therefore, this model is applicable for target-oriented in therapeutic decision making and prognostic evaluation.

Notably, in this study, some of cases with single genetic signature also carry mutations commonly identified in other B cell malignancies. For those carrying single *MYC*-trans signatures, 8 of 20 cases were affected by *ID3*-*TCF3*-*CCND3* pathway mutations, which were prevalent in Burkitt lymphoma (BL) [27–29]. For cases with single *BCL2*-trans signature, a significant proportion (21/23, 91.3%) of cases harbor gene mutations in chromatin modification, including *KMT2D*, *CREBBP*, *EP300*, and *EZH2,* and several other signaling pathways (*STAT6*, *SOCS1*, *TNFRSF14*) which were similar to the genetic feature described in follicular lymphoma [30]. In addition, for cases with single *BCL6*-trans signature, the mutations in several genes were also frequently determined in marginal zone lymphoma [31–33], including *NOTCH2* (14.6%, 6/41), *KLF2* (34.1%, 14/41), *TNFAIP3* (19.5%, 8/41), and *FAS* (17.1%, 7/41) mutations.

It should be noted that, limited by gene panel and sample size, the genetic signatures of certain cases might be mislabeled by a RF prediction algorithm, and some other important genetic signatures might remain undiscovered in our study. Several other genetic alterations have already been revealed to be of potential importance in understanding the mechanism of pathogenesis, classification, therapeutic guidance, and prognosis evaluation but were not included in our set of genetic features (Supplementary Table S6). Additionally, due to the single-center nature of our high-throughput sequencing study, current study lack external data to further support our theories. However, we did aim to validate our model in an expanded-scale multi-centered study in future exploration. Considering the DLBCL genetic diversities among different human races, we believed that future research including multiple populations would provide more consolidated evidences. Our future work will focus on undertaking a multiplatform analysis of genetic features on expanding-scale cohort, so as to promoting the lymphoma signature landscape description and to facilitate the precise determination of genetic signature.

## Conclusion

Unlike mutually exclusive molecular sub-classification, our observations supported novel insight into understanding complex genetic features by identifying the status of several non-mutually exclusive clustered genetic fingerprints. The identification of genetic signatures was also helpful for disease classification, but it was also expected to reveal actionable targets for novel therapy development and precise prognostic evaluation.

## Supplementary information

**Additional file 1: Table S1.** Patient characteristics and genetic variants information.

**Additional file 2: Table S2**. Gene list of our targeted NGS panel

**Additional file 3: Table S7**. Top 50 frequent variants included in the in-house curated blacklist.

**Additional file 4:.** Supplementary Appendix

**Additional file 5: Table S3**. Variant list

**Additional file 6: Figure S1.** The mutation pattern in 283 DLBCL. The mutational heatmap indicated the genetic alterations in 239 de novo DLBCL, NOS, 25 relapsed DLBCL after R-CHOP or CHOP-like chemotherapy, and 19 tFL, ranked by their mutational frequencies in all cases. Color code by genetic alteration type: missense mutation or in-frame deletion/insertion (blue), truncating mutation, splice donor/acceptor site mutation, or copy number loss in *TP53* (red), SHM (yellow), translocation (orange), and nondetected (gray). COO classification was also indicated above the mutational heatmap. To the right of the mutational heatmap, the frequency of genetic alterations along with the negative log_10_*P* value of the significance between de novo cases and relapsed/transformed cases are listed.

**Additional file 7: Table S4**. Binary matrix of the status of one-hot encoded genetic alterations, initial defined genetic signatures, and final convergent genetic signatures

**Additional file 8: Table S5**. Multivariate analysis including our set of four signatures and IPI score using enter method in cox proportional hazard regression model

**Additional file 9 Table S6**. Genetic alterations had been revealed to be of importance but not included in this study.

## Data Availability

The clinical and genetic variants data analyzed in this study are provided in this article and in supplementary files. The raw datasets analyzed during the current study are available from the corresponding author on reasonable request.

## Abbreviations

DLBCL: Diffuse large B-cell lymphoma
CN: Copy Number
RF: Random Forest
ABC: Activated B-cell-like
NGS: Next-generation sequencing
SV: Structure variations
PCNSL: Primary central nervous system lymphoma
PMBL: Primary mediastinal large B-cell lymphoma
PCDLBCL-LT: Primary cutaneous DLBCL, leg type
HGBL, NOS: High-grade B-cell lymphoma, not otherwise specified
HGBL-DH/TH: HGBL with *MYC* and *BCL2* and/or *BCL6* translocations ()
MHG: Molecular high-grade
tFL: Transformed follicular lymphoma
FFPE: Formalin-fixed paraffin embedded
IHC: Immunohistochemistry
FISH: Fluorescent in situ hybridization
OS: Overall survival,
PFS: Progression-free survival
CDS: Coding sequences
AID: Activation-induced cytidine deaminase
SHM: Somatic hypermutation
CLL: Chronic lymphocytic leukemia

## References

[1] Swerdlow SH, Campo E, Pileri SA, Harris NL, Stein H, Siebert R (2016). The 2016 revision of the World Health Organization classification of lymphoid neoplasms. Blood..

[2] Coiffier B (2007). Rituximab therapy in malignant lymphoma. Oncogene..

[3] Reddy A, Zhang J, Davis NS, Moffitt AB, Love CL, Waldrop A (2017). Genetic and Functional Drivers of Diffuse Large B Cell Lymphoma. Cell.

[4] Schmitz R, Wright GW, Huang DW, Johnson CA, Phelan JD, Wang JQ (2018). Genetics and pathogenesis of diffuse large B-cell lymphoma. N Engl J Med.

[5] Chapuy B, Stewart C, Dunford AJ, Kim J, Kamburov A, Redd RA (2018). Molecular subtypes of diffuse large B cell lymphoma are associated with distinct pathogenic mechanisms and outcomes. Nat Med.

[6] Alizadeh AA, Eisen MB, Davis RE, Ma C, Lossos IS, Rosenwald A (2000). Distinct types of diffuse large B-cell lymphoma identified by gene expression profiling. Nature..

[7] Gonzalez-Aguilar A, Idbaih A, Boisselier B, Habbita N, Rossetto M, Laurenge A (2012). Recurrent mutations of MYD88 and TBL1XR1 in primary central nervous system lymphomas. Clin Cancer Res.

[8] Mottok A, Hung SS, Chavez EA, Woolcock B, Telenius A, Chong LC (2019). Integrative genomic analysis identifies key pathogenic mechanisms in primary mediastinal large B-cell lymphoma. Blood..

[9] Koens L, Zoutman WH, Ngarmlertsirichai P, Przybylski GK, Grabarczyk P, Vermeer MH (2014). Nuclear factor-kappaB pathway-activating gene aberrancies in primary cutaneous large B-cell lymphoma, leg type. J Investigative Dermatol.

[10] Bouska A, Bi C, Lone W, Zhang W, Kedwaii A, Heavican T (2017). Adult high-grade B-cell lymphoma with Burkitt lymphoma signature: genomic features and potential therapeutic targets. Blood..

[11] Ennishi D, Jiang A, Boyle M, Collinge B, Grande BM, Ben-Neriah S (2019). Double-hit gene expression signature defines a distinct subgroup of germinal center B-cell-like diffuse large B-cell lymphoma. J Clin Oncol.

[12] Sha C, Barrans S, Cucco F, Bentley MA, Care MA, Cummin T (2019). Molecular high-grade B-cell lymphoma: defining a poor-risk group that requires different approaches to therapy. J Clin Oncol.

[13] Hans CP, Weisenburger DD, Greiner TC, Gascoyne RD, Delabie J, Ott G (2004). Confirmation of the molecular classification of diffuse large B-cell lymphoma by immunohistochemistry using a tissue microarray. Blood..

[14] Rimsza LM, Wright G, Schwartz M, Chan WC, Jaffe ES, Gascoyne RD (2011). Accurate classification of diffuse large B-cell lymphoma into germinal center and activated B-cell subtypes using a nuclease protection assay on formalin-fixed, paraffin-embedded tissues. Clin Cancer Res.

[15] DAWSON IM, CORNES JS, MORSON BC (1961). Primary malignant lymphoid tumours of the intestinal tract. Report of 37 cases with a study of factors influencing prognosis. Br J Surg.

[16] Cheson BD, Fisher RI, Barrington SF, Cavalli F, Schwartz LH, Zucca E (2014). Recommendations for initial evaluation, staging, and response assessment of Hodgkin and non-Hodgkin lymphoma: the Lugano classification. J Clin Oncol.

[17] Gebauer N, Bernard V, Feller AC, Merz H (2013). ID3 mutations are recurrent events in double-hit B-cell lymphomas. Anticancer Res.

[18] Panea RI, Love CL, Shingleton JR, Reddy A, Bailey JA, Moormann AM (2019). The whole-genome landscape of Burkitt lymphoma subtypes. Blood..

[19] Okosun J, Wolfson RL, Wang J, Araf S, Wilkins L, Castellano BM (2016). Recurrent mTORC1-activating RRAGC mutations in follicular lymphoma. Nat Genet.

[20] Clipson A, Wang M, de Leval L, Ashton-Key M, Wotherspoon A, Vassiliou G (2015). KLF2 mutation is the most frequent somatic change in splenic marginal zone lymphoma and identifies a subset with distinct genotype. Leukemia..

[21] Wang K, Li M, Hakonarson H (2010). ANNOVAR: functional annotation of genetic variants from high-throughput sequencing data. Nucleic Acids Res.

[22] Khodabakhshi AH, Morin RD, Fejes AP, Mungall AJ, Mungall KL, Bolger-Munro M (2012). Recurrent targets of aberrant somatic hypermutation in lymphoma. Oncotarget..

[23] de Miranda NFCC, Georgiou K, Chen L, Wu C, Gao Z, Zaravinos A (2014). Exome sequencing reveals novel mutation targets in diffuse large B-cell lymphomas derived from Chinese patients. Blood..

[24] Karube K, Enjuanes A, Dlouhy I, Jares P, Martin-Garcia D, Nadeu F (2018). Integrating genomic alterations in diffuse large B-cell lymphoma identifies new relevant pathways and potential therapeutic targets. Leukemia..

[25] Puente XS, Pinyol M, Quesada V, Conde L, Ordonez GR, Villamor N (2011). Whole-genome sequencing identifies recurrent mutations in chronic lymphocytic leukaemia. Nature..

[26] Jardin F, Pujals A, Pelletier L, Bohers E, Camus V, Mareschal S (2016). Recurrent mutations of the exportin 1 gene (XPO1) and their impact on selective inhibitor of nuclear export compounds sensitivity in primary mediastinal B-cell lymphoma. Am J Hematol.

[27] Schmitz R, Young RM, Ceribelli M, Jhavar S, Xiao W, Zhang M (2012). Burkitt lymphoma pathogenesis and therapeutic targets from structural and functional genomics. Nature..

[28] Love C, Sun Z, Jima D, Li G, Zhang J, Miles R (2012). The genetic landscape of mutations in Burkitt lymphoma. Nat Genet.

[29] Lopez C, Kleinheinz K, Aukema SM, Rohde M, Bernhart SH, Hubschmann D (2019). Genomic and transcriptomic changes complement each other in the pathogenesis of sporadic Burkitt lymphoma. Nat Commun.

[30] Krysiak K, Gomez F, White BS, Matlock M, Miller CA, Trani L (2017). Recurrent somatic mutations affecting B-cell receptor signaling pathway genes in follicular lymphoma. Blood..

[31] Rossi D, Trifonov V, Fangazio M, Bruscaggin A, Rasi S, Spina V (2012). The coding genome of splenic marginal zone lymphoma: activation of NOTCH2 and other pathways regulating marginal zone development. J Exp Med.

[32] Spina V, Khiabanian H, Messina M, Monti S, Cascione L, Bruscaggin A (2016). The genetics of nodal marginal zone lymphoma. Blood..

[33] Zhang Q, Siebert R, Yan M, Hinzmann B, Cui X, Xue L (1999). Inactivating mutations and overexpression of BCL10, a caspase recruitment domain-containing gene, in MALT lymphoma with t (1;14)(p22;q32). Nat Genet.

