# Supplementary material for: Novel bioinformatic classification system for genetic signatures identification in diffuse large B-cell lymphoma
Source: BMC Cancer. 2020 Jul 31;20:714. doi: 10.1186/s12885-020-07198-1 (PMC7393908; doi:10.1186/s12885-020-07198-1)
Supplement: Supplementary file 4 — Additional file 4:. Supplementary Appendix [file 12885_2020_7198_MOESM4_ESM.docx]

**Supplementary Appendix**

This appendix has been provided by the authors to give readers additional information about their work.

**Machine learning-based algorithm**

We initially defined four non-mutual exclusive genetic signatures with prevalent specialized genetic alterations:

1. *MYC*-trans signature, with *MYC* translocation (n = 54)
2. *BCL2*-trans signature, with *BCL2* translocation (n = 59)
3. *BCL6*-trans signature, with *BCL6* translocation (n = 91)
4. MC signature, with *MYD88*^L265P^ and/or *CD79B*^Y196^ mutations (n = 72)

Inspired by the study conducted by R. Schmitz *et al*.[^1^](#_ENREF_1), we aimed to evolve and maximize each genetic signature with our set of genetic features while appropriately maintaining the pattern suggested by initial genetic signature. We developed a machine learning algorithm to cope with such issue as follows:

Given a *m*-dimensional binary feature vector x and *n*-dimensional binary label vector y, where x ∈ {0, 1} *^m^* and y ∈ {0, 1} *^n^*. In other words, we had a feature matrix X and a full label matrix Y,

X = [x_1_, x_2_, … , x*_k_*]^T^, where X ∈ {0, 1} *^k^* ^×^ *^m^*

Y = [y_1_, y_2_, … , y*_k_*]^T^, where Y ∈ {0, 1} *^k^* ^×^ *^n^*

Let X*_ij_* = 1 be the *i*-th case had the *j*-th feature and Y*_ij_* = 1 be the *i*-th case had the *j*-th label.

We initially included mutation status in all 46 targeted genes and *MYC*/*BCL2*/*BCL6* translocations as candidate features. We also took COO subtype into consideration since it could complement and indicate an overall effect of other undetected genetic alterations. To create a binary column for each feature, gene mutations were subsequently split and encoded using a one-hot encoding scheme based on three non-mutual exclusive type of mutation status (non-truncating mutation, truncating mutation, or somatic hypermutation). In addition, *MYD88*^L265P^/*CD79B*^Y196^ were separated from other *MYD88*/*CD79B* non-truncating mutations. Finally, we got a 91-dimensional feature vector (**Supplementary Table S4**). Among them, main features including *MYC* translocation, *BCL2* translocation, *BCL6* translocation, *MYD88*^L265P^, and *CD79B*^Y196^ mutations that initially defined four genetic signatures consisted of feature vector p ∈ {0, 1} *^a^*, and other features consisted of feature vector q ∈ {0, 1} *^b^* where *a* + *b* = *m*, and similarly we can get matrix P ∈ {0, 1} *^k^* ^×^ *^a^* and Q ∈ {0, 1} *^k^* ^×^ *^b^*.

On the other hand, although a 4-dimensional label vector y that represented four genetic signatures were initially defined as mentioned above, here, we intended to extend the conception of genetic signatures. In this study, the full label matrix Y was not available and instead we were only given a label occurrence matrix O ∈ {0, 1} *^k^* ^×^ *^n^* where O*_ij_* = 1 meant the *i*-th case was observed having the *j*-th label. While when O*_ij_* = 0, the underlying label Y*_ij_* had either an unobserved *j*-th label or did not have a *j*-th label. Observed labels were valued entirely according to the initial definition mentioned above. Our goal was to get a predictive label matrix Ŷ ∈ {0, 1} *^n^* ^×^ *^k^* from {X, O} to approximate Y.

Obviously, such problem could be considered as a semi-supervised learning problem, which could be solved by several methods based on the smoothness assumption, the cluster assumption, and the manifold assumption. Label propagation algorithm (LPA) and its derivative methods are classic network community detections to assigns labels to previously unlabeled data points, using iterative methods. Our problem could have been transformed into a *semi-supervised weak-label learning*{Dong, 2018 #28} but it would bring more algorithmic error since our set of genetic features might be uncompleted. Thus, considering the general better perform of ensemble learning, we developed a method based on iterative random forest (RF) algorithms as follows:

1. To eliminate propagation constraint, instead of {X, O}, we started with a training set {Q, O} containing all *i*-th cases, ∀*i* = 1, . . . , k. An RF model was trained on preliminary training set and a leave-one-out (LOO) cross validation prediction was also applied within {Q, O}, thereby generating a preliminary label prediction matrix R^0^ ∈ {0, 1} *^k^* ^×^ *^n^*. Given three matrixes U, V, and W with the same dimension, if ∀U*_ij_* = max(V*_ij_*, W*_ij_*), we denoted U = max(V, W). Thus, we could also get a matrix O^1^, where O^1^ = max(O, R^0^). Here, O^1^ – O was the additionally extended label matrix predicted by RF model.
2. Next, another RF model with optimized parameters was trained on {Q, O^1^}, a novel label prediction matrix R^1^ ∈ {0, 1} *^k^* ^×^ *^n^* was got by LOO cross validation prediction. Similarly, let matrix O^2^ = max(O, R^1^). And so on, RF model with identical parameters iteratively run *i* times, when R*^i^* was generated from a LOO cross validation prediction from training dataset {Q, O*^i^* ^- 1^}, and O*^i^* = max(O, R*^i^* ^- 1^). We halted the iteration at *i* = *imax*, once the density of sparse matrix O*^i^* – O*^i - 1^* was less than 0.001, or *imax* =20 if a convergence was still not obtained at the 20^th^ iteration. Here, let O*^i^* ∈ {0, 1} *^n^* ^×^ *^k^* be the final label prediction matrix of our cohort if the iterative RF model obtained a convergence.

Finally, we halted the iteration at *i* = *7*, since the density of sparse matrix O^7^ – O^6^ was 7.31×10^-4^ (< 0.001). The final extended label matrix of genetic signatures among cases was listed in **Supplementary Table S4** and the statistics were as follows:

1. *MYC*-trans signature (n = 62)
2. *BCL2*-trans signature (n = 69)
3. *BCL6*-trans signature (n = 108)
4. MC signature (n = 115)

1 Schmitz, R. *et al.* Genetics and Pathogenesis of Diffuse Large B-Cell Lymphoma. *The New England journal of medicine* **378**, 1396-1407, doi:10.1056/NEJMoa1801445 (2018).
